# Fat Grafting Technique, A Paradigm Shift in the Treatment of Tuberous Breast

**Published:** 2018-01

**Authors:** Claudiox Claudio Silva-Vergara, Joan Fontdevila, Omar Weshahy

**Affiliations:** Hospital Clinic of Barcelona. University of Barcelona, Barcelona, Spain

**Keywords:** Tuberous breast, Lipofilling, Fat graft

## Abstract

**BACKGROUND:**

The tuberous breast syndrome is a condition that affects young women and can bring on serious disorders of self-esteem. There are numerous surgical techniques to correct this condition, but most of them include the use of breast implants to replace lack of volume. Nowadays, patients are increasingly becoming more demanding to get a definitive solution with minimal aesthetic sequelae.

**METHODS:**

We present a series of 11 patients with tuberous breast deformities treated with lipofilling technique. Fat harvest was performed by conventional lipoaspiration with 3 mm cannulas, centrifuged at 2000 rpm for 2 minutes and injected in the breast were was needed with 1.9 mm blunt cannulas.

**RESULTS:**

The patient’s average age was 24 year old with a BMI of 23.4 kg/m^2^. Volumes between 80 and 250 ml per breast were injected in every session, requiring a total volume of 413 ml per breast. Most patients required up to two procedures to achieve the complete breast correction. The mean follow-up was 29.7 months. All patients had good aesthetics results with minimal complications.

**CONCLUSION:**

Natural breast mound with excellent cosmetic result can be achieved with fat grafting. Fat grafting main advantage is to respond to physiological changes in weight over time. In addition, lipofilling do not carry the inherent complications of implants. We strongly believe it is a safe and easy technique to reproduce in properly selected patients and with minimal morbidity it can cause the best natural result.

## INTRODUCTION

The tuberous breast syndrome is the result of a complex series of defects with various degrees of expression that becomes evident at puberty in young women. It is characterized by several morphologic alterations bilateral or unilateral, with a wide spectrum of expression. Resulting in a small breast, cylindrical shape with a deficient diameter of the mammary base, hypoplasia especially of the lower quadrants, abnormal elevation of the inframammary fold, breast tissue herniation into the areolar region; and sometimes increased areolar diameter.^1^^,^^2^ The exact incidence of the tuberous breast is unknown and is probably impossible to ascertain because many women who have mild degrees of deformity may not seek help or be aware of that deformity exists.^1^

Several surgical approaches have been described to correct the tuberous breast deformity since Rees and Aston first described the correction of the tuberous breast in 1976. Most of them require a combination of breast remodeling techniques and the employment of implants.^2^ Lately a new technique has been gaining support since Coleman reported on the benefits of lipoaugmentation as an auxiliary technique in aesthetic breast reconstruction.^3^ Following this pathway, we present 11 patients in who we opt for lipofilling technique for tuberous breast remodeling.

## MATERIALS AND METHODS

Between the years 2008-2014, a total of 11 patients with 19 tuberous breast deformities underwent autologous fat grafting in our institution for aesthetic improvement. Tuberous breasts were classified according to Grolleau *et al.* classification system,^4^ where type I is hypoplasia of the medial lower quadrant, type II is hypoplasia of both lower quadrants and type III is hypoplasia of the four quadrants with severe breast constriction. 

**Fig. 1:**
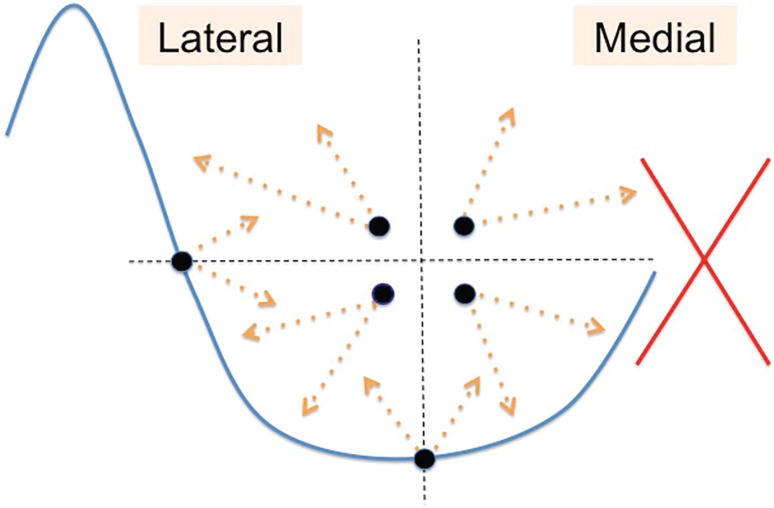
The dots indicate the place through which lipofilling is performed. Note that the medial side is avoided to reduce the risk of unaesthetic scars.

Patients were drawn in a standing position. The breast was divided into 4 quadrants for better control over the volumes injected and the new mammary fold was painted in a lower position. Donor areas used were meanly abdomen and flanks, but depend basically on patient´s adipose tissue distribution. We infiltrated with a saline solution containing epinephrine and harvesting was performed by conventional lipoaspiration with 3 mm cannulas. To minimize adipocyte damage, fat was aspirated at 40 kPa with a vacuum pump through an intermediary 400 ml modified drainage bottle as fat storage.^5^ Fat was then centrifuged at 2000 rpm (400 G) for 2 min to obtain purified fat with almost nonexistent oily supernatant without compromising efficiency of the graft. Then this was injected using 10 ml syringes with 1.9 mm blunt cannulas of 9 cm long through four punctures around the areola rim and one at the anterior axillary line (Figure 1).

## RESULTS

The mean age was 24 years old and BMI was 23.4 kg/m^2^. The breasts were distributed according Grolleau`s classification: 3 were type I (15.8%), 13 were type II (68.4%) and 3 were considered type III (15.8%). There was some sort of asymmetry in most patients. Areolar prolapse was noted on 5 tuberous breast (26.3%). There were no other anomalies present, and no family history of breast malformation was recorded. Each patient was evaluated individually and the numbers of fat grafting procedures were performed according to the particular needs of each one. 

**Fig. 2:**
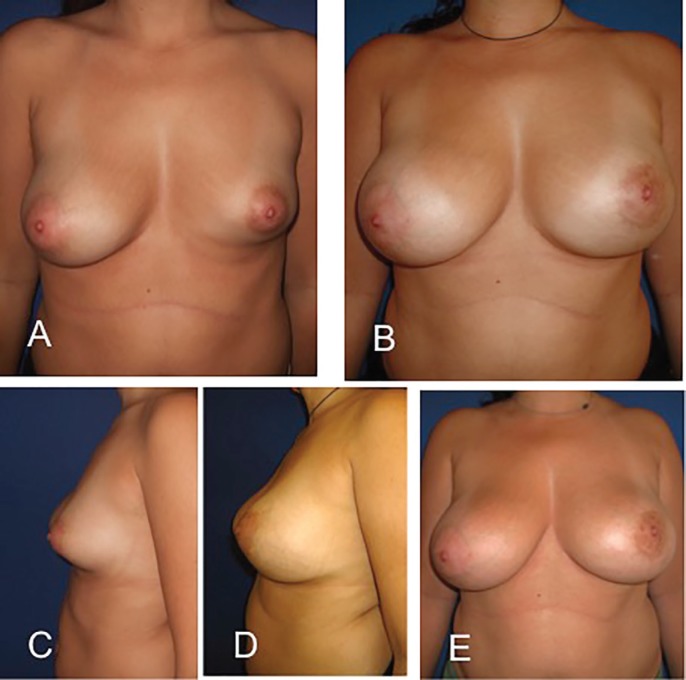
A and C, Preoperative views of a 30-year-old woman (patient 4). She had 3 fat grafting procedures with a total of 550 ml. B and D, Postoperative view after three sessions of breast lipofilling seven months after the last procedure (injections of 240 ml, 160 ml and 150 ml, respectively). Picture E was after 51 months of follow up, patient has gained weight and the breast size grows naturally over time.

**Fig. 3:**
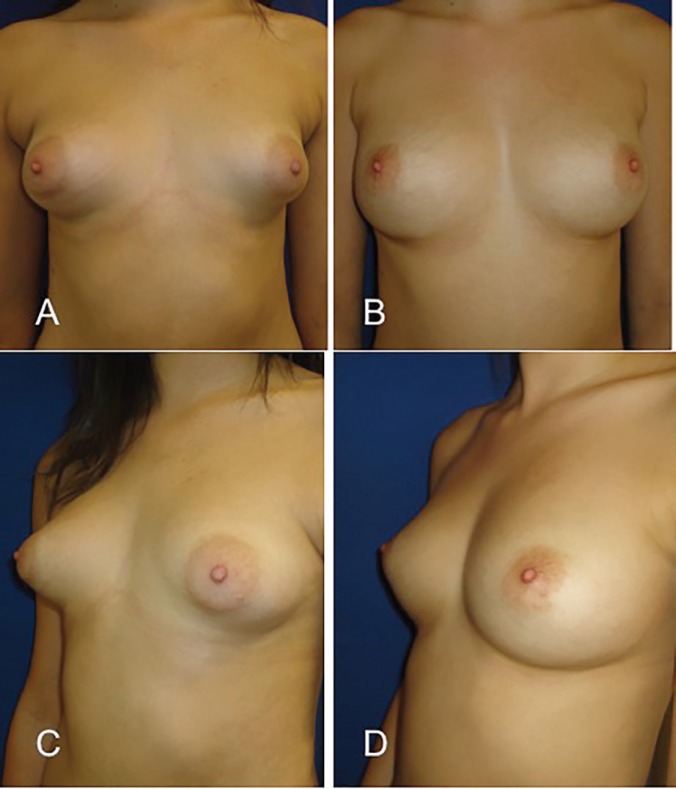
A and C, Preoperative views of a 17-year-old woman with bilateral tuberous breast (patient 3). B and D, Postoperative view after two sessions of breast lipofilling. The benefit over the areola are evident, areolar herniation has improved, the skin is no longer tense and areolar diameter decreases only with the contribution of fat graft that releases the tight lower breast pole. No areola reduction surgery has been made.

**Fig. 4:**
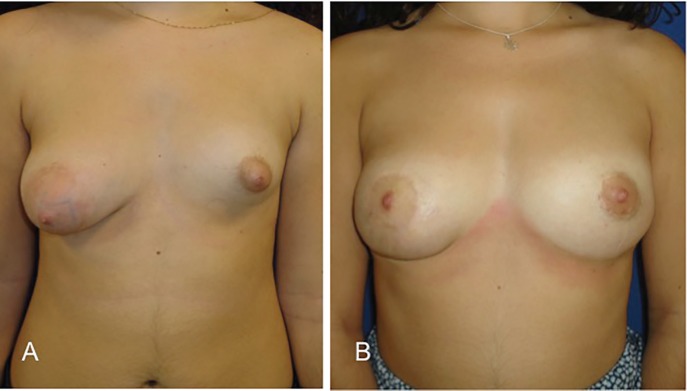
Preoperative views of a 19-year-old woman with marked breast asymmetry (patient 5). Postoperative view shows a periareolar mastopexy on the right breast and two lipofilling sessions in the left breast at 35 months follow-up.

**Fig. 5:**
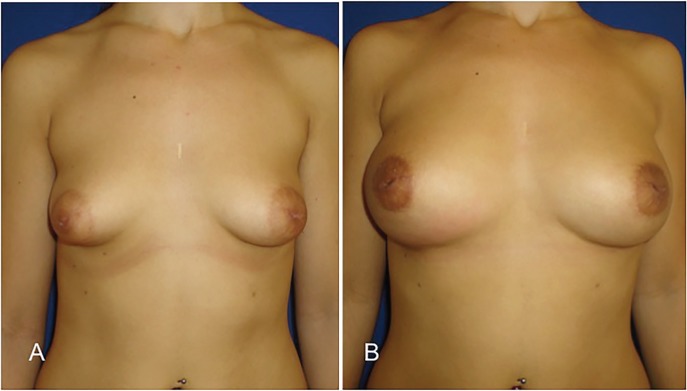
This 22-year-old patient needed 3 procedures and a total of 520 ml in each breast (patient 6). The right photograph is at 25 months after the first lipofilling. A 7mm oil cyst was drained with a fine needle at medical office without tarouble.

**Fig. 6:**
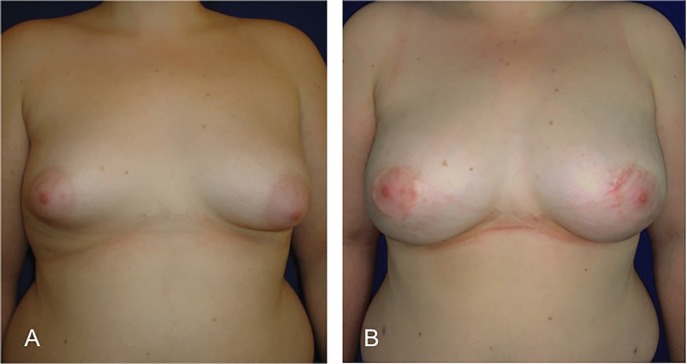
Fat graft is an ideal technique for patients with obesity as patient 9. She had a 33.6 BMI and she required 2 lipofilling procedures to complete the bilateral tuberous breast correction.

Two patients required only 1 procedure, seven patients required 2 procedures and two patients required a total of 3 procedures to obtain a suitable volume (Figure 2-6). Volumes of fat infiltrated in each breast varied from 80 to 250 ml per procedure (average 210 ml). There were no surgical complications such as seroma, hematomas or infections of neither the donor area nor the breast. Mild common ecchymosis was observed. In 4 patients an oil cyst had been detected with ultrasound but only in one of them the patient palpated it. In 2 patients the oil cyst was drained with a needle without any difficulty at the medical office. Patients’ characteristics and results are depicted in Table 1.

**Table 1:** Characteristics of patients and lipofilling data

**Case**	**Age (years)**	**BMI (kg/m** ^2^ **)**	**Tuberous breast (n)**	**Grolleau classification**	**Sessions** **of LF (n) **	**Fat graft per breast (ml) **	**Complications**	**Follow-up (months)**
1	22	21.4	2	II	2	420	-	65
2	25	22.1	2	III	2	480	Oil Cyst 5mm	39
3	17	20.2	2	II	2	430	Oil Cyst 8mm	38
4	30	24.2	1	II	3	550	-	51
5	19	23.5	1	III	2	450	-	35
6	22	22.6	2	II	3	520	Oil Cyst 7mm	25
7	19	22.3	2	II	2	410	-	21
8	19	20.5	2	II	2	400	-	15
9	26	33.6	2	II	2	440	Oil Cyst 2mm	17
10	39	25.1	1	I	1	250	-	13
11	24	21.7	2	I	1	190	-	8
Mean	24	23.4			2.0	413		29.7

LF: Lipofilling

## DISCUSSION

Since Coleman popularized lipofilling technique,^3^ it has become a recognized therapeutic tool for soft tissue augmentation. A variety of independent investigators suggest that fat grafting to the breast is a procedure that can yield natural, long-term results.^6^^,^^7^ The exact pathologic mechanisms underlying the development of the tuberous breast deformity are still unknown so far.^8^ However, recent evidence shows that the etiopathology may be a result of collagen deposition alterations and these facts appear to involve nearly all the breast stroma, not only the superficial fascia, making tuberous breast a complex and multilayer malformation.^9^ That why lipofilling probably play´s a key role in the treatment of this anomaly, it modifies globally the breast parenchyma to attain a better aesthetic result.

In treated patients we found an unexpectedly easy correction of the lower quadrants by increased volume and better definition of inframammary fold. All this happened without need of any implant, glandular flaps or aggressive deep radial incisions across the gland as classical techniques do. As well, this avoided possible complications such as double bubble deformity, capsular contracture and the risk of prosthetic extrusion for excessive manipulation of the gland. Additionally, we observed a softening of the breast, coinciding with previous observations in interstitial tissues when receiving fat grafting.^10^ Furthermore, the postoperative course is much more comfortable for the patient with more predictable results. Especially that behaves naturally and in harmony with patients weight changes over the years (Figure 2). 

It is important to note that injection under the nipple-areola complex must be avoided in cases of nipple herniation as this may worsen the abnormality. However, by reducing the constricted area of the lower pole with lipofilling, immediately improves the areola herniation without need in most of cases of any additional surgery (Figure 3). All patients were satisfied with the cosmetics results. So far, none of our patients has had suspicious results in the monitoring reports of breast imaging. Liponecrosis or oil cysts detected on routine breast screening are easily recognized as benign lesions by trained radiologists.

We recommend performing lipofilling procedures with at least a 6 months interval. At this time the tissue has already been fully integrated and inflammation/fibrosis disappeared. One aspect that could be criticized of this technique is that usually requires more than one procedure to complete a satisfactory cosmetic result. However, if we consider the long-term results, it´s very hard to believe that a patient with breast implants placed at age of 20, will not require in the course of her life of any surgical revision. Unfortunately, this technique does not work for all patients, very thin ones who have no suitable donor sites are not able to benefit from lipofilling.

Our preliminary results using lipofilling to treat young patients with tuberous breast are encouraging. Better, natural and more reliable results can be achieved with minimal scarring and without need of any prosthetic implant. We believe lipofilling technique should be considered as an alternative of first option for correction of breast anomalies such as tuberous breast in patients with suitable donor sites.
